# Assessment of risk factors related to healthcare-associated methicillin-resistant *Staphylococcus aureus *infection at patient admission to an intensive care unit in Japan

**DOI:** 10.1186/1471-2334-11-303

**Published:** 2011-11-01

**Authors:** Kazuma Yamakawa, Osamu Tasaki, Miyuki Fukuyama, Junichi Kitayama, Hiroki Matsuda, Yasushi Nakamori, Satoshi Fujimi, Hiroshi Ogura, Yasuyuki Kuwagata, Toshimitsu Hamasaki, Takeshi Shimazu

**Affiliations:** 1 Department of Emergency and Critical Care, Osaka General Medical Center, Osaka, Japan; 2 Department of Traumatology and Acute Critical Medicine, Osaka University Graduate School of Medicine, Osaka, Japan; 3 Department of Biomedical Statistics, Osaka University Graduate School of Medicine, Osaka, Japan

## Abstract

**Background:**

Healthcare-associated methicillin-resistant *Staphylococcus aureus *(HA-MRSA) infection in intensive care unit (ICU) patients prolongs ICU stay and causes high mortality. Predicting HA-MRSA infection on admission can strengthen precautions against MRSA transmission. This study aimed to clarify the risk factors for HA-MRSA infection in an ICU from data obtained within 24 hours of patient ICU admission.

**Methods:**

We prospectively studied HA-MRSA infection in 474 consecutive patients admitted for more than 2 days to our medical, surgical, and trauma ICU in a tertiary referral hospital in Japan. Data obtained from patients within 24 hours of ICU admission on 11 prognostic variables possibly related to outcome were evaluated to predict infection risk in the early phase of ICU stay. Stepwise multivariate logistic regression analysis was used to identify independent risk factors for HA-MRSA infection.

**Results:**

Thirty patients (6.3%) had MRSA infection, and 444 patients (93.7%) were infection-free. Intubation, existence of open wound, treatment with antibiotics, and steroid administration, all occurring within 24 hours of ICU admission, were detected as independent prognostic indicators. Patients with intubation or open wound comprised 96.7% of MRSA-infected patients but only 57.4% of all patients admitted.

**Conclusions:**

Four prognostic variables were found to be risk factors for HA-MRSA infection in ICU: intubation, open wound, treatment with antibiotics, and steroid administration, all occurring within 24 hours of ICU admission. Preemptive infection control in patients with these risk factors might effectively decrease HA-MRSA infection.

## Background

Healthcare-associated methicillin-resistant *Staphylococcus aureus *(HA-MRSA) infection in critically ill patients is associated with prolonged intensive care unit (ICU) stay, increased medical cost, and high mortality [1,2]. Furthermore, patients in the ICU have an increased susceptibility to HA-MRSA infections [3,4]. Special risk factors make such patients temporarily immunocompromised: normal host defense mechanisms are often disrupted by multiple invasive devices, impaired by underlying disease, and reduced by medical interventions and medications. Overall, intrinsic together with extrinsic risk factors make the ICU patient extremely vulnerable to HA-MRSA infections. Therefore, control of HA-MRSA transmission and infection in the ICU is a serious concern.

Although most patients in the ICU are critically ill, to perform infection control precautions for all ICU patients would place an additional burden on medical staff and might result in insufficient infection control. If the patients at high risk of MRSA infection can be detected on ICU admission, it may be possible to focus preemptive infection control measures on such patients and lessen the workload of the ICU medical staff. The purpose of this study was to clarify the risk factors of HA-MRSA infection in our combination medical, surgical, and trauma ICU and to determine the group of patients we should target for preemptive infection control.

## Methods

### Patient population

This was a prospective cohort study conducted from April 2009 to March 2010, during which time 1284 consecutive patients were admitted to the ICU of Osaka General Medical Center, Japan. Of these patients, 493 consecutive patients who stayed in the ICU for more than 2 days were included in the present study. Nineteen patients were excluded from the analysis as being MRSA-positive on admission because MRSA was detected by the first screening culture within 2 days after ICU admission. Thus, a total of 474 patients comprised the study group.

This study followed the principles of the Declaration of Helsinki. The conduction of this study was approved by the institutional review board at Osaka General Medical Center. The board waived the need for informed consent because we have taken the samples for surveillance not purely for the purpose of this study.

### Infection control policy

The Osaka General Medical Center is a 768-bed, acute, tertiary referral hospital. The 18-bed ICU is both a medical and surgical ICU with large numbers of trauma patients. Standard precautions, such as hand hygiene with alcohol gel or soap before and after patient care, are used for all patients, regardless of multidrug-resistant organisms (MDRO) colonization status. In addition, MDRO-colonized patients are placed in isolation, and contact precautions, such as the wearing of disposable gowns, gloves, and masks during the care of these patients, are performed. Contact precautions were also applied to the patients transferred from other hospitals until MRSA status could be proven to be negative by surveillance culture.

### Data collection

We performed surveillance culture of sputum, nasal excretions, and urine when patients were enrolled. Nasal, pharyngeal, and wound specimens were obtained with cotton-tipped sticks. Surveillance cultures were continued once every week while the patients remained in the ICU. Other clinical cultures were performed when needed.

Clinical samples were processed according to routine microbiology procedures. Gram-positive cocci were tested for catalase production, and catalase-positive colonies were then tested for coagulase. Any coagulase-positive colony was subcultured onto non-selective blood agar for identification and susceptibility testing. Antibiotic susceptibility pattern was determined using the Vitek 1 system (Sysmex bioMérieux Co., Ltd., Tokyo, Japan), following the criteria of the Clinical and Laboratory Standards Institute. Genotypic analysis of the strains was not performed.

The patients whose surveillance or clinical cultures became positive for MRSA after enrollment were defined as "HA-MRSA acquisition." Acquisition included apparent infection and/or colonization by MRSA. Infection was diagnosed according to the Centers for Disease Control and Prevention National Nosocomial Infections Surveillance System definitions [5]. We evaluated the rate of healthcare acquisition and/or apparent infection with MRSA. Follow-up was for the duration of the ICU stay.

We assessed a total of 11 candidate prognostic variables possibly related to MRSA infection: baseline characteristics (age and sex), severe sepsis, severe trauma, existence of open wound, history of emergency operation, intubation, insertion of central venous catheters (CVCs), antibiotics administration, steroid administration, and transfer from another hospital. Detailed definitions of each variable are shown in Table 1. Data on all variables used in this study were obtained within 24 hours of ICU admission to predict the risk of infection in the early phase of ICU stay.

**Table 1:** Definitions of 11 candidate prognostic variables possibly related to MRSA infection

Variable	Definition
Age	Years
Sex	Male or female
Severe sepsis	According to the criteria of the ACCP/SCCM consensus conference committee
Severe trauma	AIS score greater than 2 for at least one region
Open wound	Existence of open wound within 24 hrs of ICU admission
Emergency operation	Emergency operation within 24 hrs of ICU admission
Intubation	Intubation within 24 hrs, except extubation within 24 hrs of ICU admission
CVCs	Insertion of central venous catheters within 24 hrs of ICU admission
Antibiotics	Antibiotics administration within 24 hrs of ICU admission
Steroid	History of steroid use or steroid administration within 24 hrs of ICU admission
Transferred	Transferred from another hospital

MRSA: methicillin-resistant *Staphylococcus aureus*; ACCP/SCCM: American College of Chest Physicians/Society of Critical Care Medicine; AIS: abbreviated injury scale; CVCs: central venous catheters.

### Statistical analysis

The most important prognostic variables were selected as follows. First, univariate logistic regression analysis was conducted to find potential prognostic variables; variables with a *P*-value > 0.20 were excluded. Second, stepwise multivariate logistic regression analysis was performed to identify independent risk factors for HA-MRSA infection. Finally, we examined sensitivity and specificity for HA-MRSA infection of each combination of risk factors. A *P*-value of < 0.05 was considered to be statistically significant. Statistical analyses were performed with SPSS version 17.0 for Windows (SPSS Inc., Chicago, IL).

## Results

**Figure 1:**
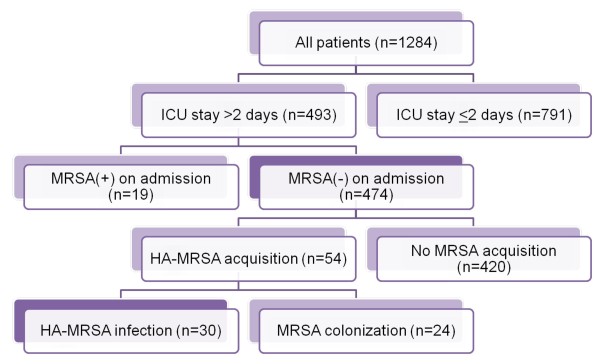
**Flowchart showing overall incidence of acquisition and infection with healthcare-associated MRSA in the present study**. MRSA: methicillin-resistant *Staphylococcus aureus*; ICU: intensive care unit.

The overall incidence of HA-MRSA infection is shown in Figure 1. In a total of 474 patients negative for MRSA on ICU admission, 30 patients (6.3%) were infected with MRSA and 444 patients (93.7%) had no infection. HA-MRSA infections were classified as pneumoniae (n = 12), burn wound infection (n = 7), soft tissue infection (n = 4), surgical site infection (n = 3), bone and joint infection (n = 2), gastroenteritis (n = 1), and bloodstream infection (n = 1).

The results of univariate analysis are shown in Table 2. Seven variables were significantly associated with HA-MRSA infection: severe sepsis (n = 44), open wound (n = 42), emergency operation (n = 84), intubation (n = 252), CVCs (n = 114), treatment with antibiotics (n = 244), and steroid administration (n = 43). Because there was no apparent relation between HA-MRSA infection and the variables severe trauma (n = 91) and transfer from another hospital (n = 117), these two variables were excluded as candidates for further analysis by multivariate logistic regression. Consequently, four risk factors were selected by multivariate logistic regression: intubation, open wound, treatment with antibiotics, and steroid administration (Table 3).

**Table 2:** Results of univariate analysis of variables related to MRSA infection

Variable	*P*-value
Age	0.053
Sex	0.151
Severe sepsis	< 0.001
Severe trauma	0.552
Open wound	< 0.001
Emergency operation	0.021
Intubation	< 0.001
CVCs	< 0.001
Antibiotics	< 0.001
Steroid	0.005
Transferred	0.485

MRSA: methicillin-resistant

*Staphylococcus aureus*; CVCs: central venous catheters.

**Table 3:** Results of multivariate logistic regression analysis

Covariate	Coeff(β)	SE(β)	OR	95% CI	*P*-value
Intubation (1 or 0)	1.961	0.651	7.109	1.983 - 25.485	0.002
Open wound (1 or 0)	1.944	0.483	6.985	2.710 - 18.005	< 0.001
Antibiotics (1 or 0)	1.625	0.646	5.078	1.433 - 17.999	0.012
Steroid (1 or 0)	1.537	0.551	4.652	1.579 - 13.709	0.005

Coeff(β): coefficient; SE(β): standard error of coefficient; OR: odds ratio; 95% CI: 95% confidential interval.

**Figure 2:**
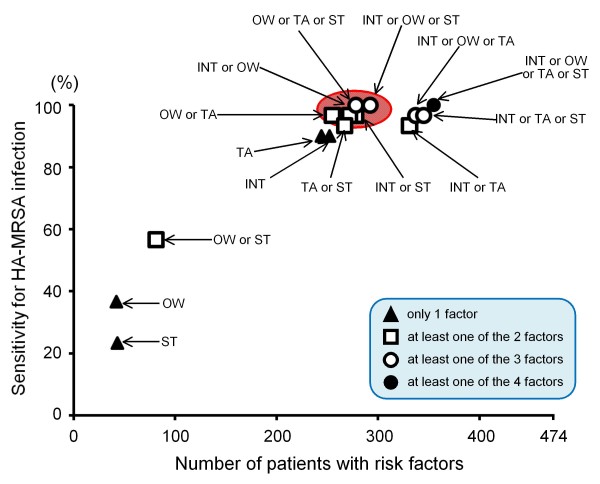
**Sensitivity for MRSA infection and number of patients with risk factors**. Sensitivity for MRSA infection is plotted according to the number of patients with at least one of the four independent risk factors identified in the present study. A closed circle indicates sensitivity and number of patients with at least one of the 4 factors, an open circle indicates sensitivity and number of patients with at least one of 3 factors, an open square indicates sensitivity and number of patients with at least one of 2 factors, and a closed triangle indicates sensitivity and number of patients with only 1 factor. The combinations circumscribed by the ellipse were the best subset of a small group of patients with the highest sensitivity for MRSA infection. MRSA: methicillin-resistant *Staphylococcus aureus*; INT: intubation; OW: open wound; TA: treatment with antibiotics; ST: steroid administration.

The sensitivity for occurrence of MRSA infection was plotted based on the number of patients with at least one of these four independent risk factors as shown in Figure 2. Infection control precautions are considered to be reasonable when targeting the small group of patients with the highest sensitivity for acquisition of MRSA infection, which is indicated by the ellipse in Figure 2. The factors within the ellipse are shown in Table 4. All five of these various combinations of risk factors for MRSA infection showed high sensitivity (96.7% - 100%). Additionally, the number of patients to target comprised between 53.8% and 61.6% of all patients admitted to the ICU during the study period. As a result, if we perform preemptive infection control measures on intubated patients or those with open wounds, this would cover 96.7% of the patients infected with HA-MRSA, and the number of patients with these two factors would be limited to just 57.4% of all patients admitted.

**Table 4:** Sensitivity and specificity for MRSA infection

	Sensitivity (%)	Specificity (%)	Number of patients (%)
Intubation or open wound	29/30 (96.7)	201/444 (45.3)	272/474 (57.4)
Intubation or steroid	29/30 (96.7)	200/444 (45.1)	273/474 (57.6)
Open wound or antibiotics	29/30 (96.7)	218/444 (49.1)	255/474 (53.8)
Intubation or open wound or steroid	30/30 (100)	182/444 (41.0)	292/474 (61.4)
Open wound or antibiotics or steroid	30/30 (100)	196/444 (44.1)	278/474 (58.6)

MRSA: methicillin-resistant *Staphylococcus aureus*.

## Discussion

We evaluated risk factors for infection with HA-MRSA during the ICU stay by multivariate logistic regression analysis. Because we plan to introduce preemptive infection control measures for patients at high risk of HA-MRSA infection in the near future, only the factors obtained within 24 hours of ICU admission were sampled as possible prognostic variables in this study. To our knowledge, there are no previously published data on risk factors for MRSA infection analyzed from such a point of view. Our results showed that four risk factors were independently associated with HA-MRSA infection: intubation, open wound, treatment with antibiotics, and steroid administration, all occurring within 24 hours of admission to the ICU.

Several reports concerning the risk factors of HA-MRSA infection have been published. Ibelings and coworkers reported that patients in the ICU are at high risk of MRSA infection, and patients with MRSA infections are less likely to survive than those with methicillin-sensitive *Staphylococcus aureus *[3]. In addition, several factors related to MRSA transmission and infection were reported, such as length of ICU stay [2], antibiotics administration [6,7], previous hospital stay [8], history of surgery [8], trauma patients [9], burn patients [10], presence of CVCs [11], and steroid administration [12]. Referring to these reports, we extracted 11 candidate prognostic variables that were possibly related to MRSA infection and that could be assessed within 24 hours of ICU admission. Although the length of ICU stay is strongly related to MRSA infection, this factor cannot be determined within the first 24 hours of ICU admission. Accordingly, we excluded this factor from the prognostic variables in this study. We also considered using a severity score, such as the Acute Physiology and Chronic Health Evaluation score or the Simplified Acute Physiology Score II score, as a prognostic variable. However, the complicated prediction calculation formulas of these scoring systems were not suitable in clinical practice, so severity scores were not considered in this study.

Although the risk factors detected in this study other than intubation did not contradict those of previous studies [6,7,10,12], no previously published data, to our knowledge, has focused on intubation as a risk factor for HA-MRSA infection. There were several reasons why intubation was selected as one of the independent risk factors. First, the frequency of medical staff contact is high for the intubated patient compared with that for other patients. In an intubated patient, frequent nursing care such as suctioning of secretions, oral care, and postural change are necessary. As a result, the opportunity for MRSA transmission will increase. Second, patients who require ventilatory support may be severely ill. It has been shown that severely ill patients tend to acquire MRSA infection [3].

The costs of MRSA infection in terms of added morbidity, mortality, hospital days, and hospital charges are overwhelming [1,2]. Although 'time-dependent bias' reportedly gives estimates that greatly overestimate the effect of nosocomial infection on the extra length of ICU stay [13], several studies have indicated that MRSA infections cause a significant additional length of stay or financial burden after adjustment for the factors that truly influence the length of stay [2,14,15]. The spread of MRSA occurs mainly from person to person [16]. Therefore, control of MRSA transmission and infection is of serious concern. The findings of this study should be helpful in formulating and implementing several strategies for reducing the risk of MRSA infection. If we strengthen preemptive infection control in high-risk patients, we may be able to decrease the rate of healthcare-associated infection. We found that patients with 2 or 3 risk factors included nearly 100% of MRSA-infected patients, whereas those factors were limited to about one half of all patients admitted, as shown in Table 4. We believe that the two factors of intubation and open wound are the best combination to address. The main reason is that these two factors are quite simple and obvious to detect, so that it is easy for the medical staff to judge whether the patient is positive for these factors. Conversely, it is hard to identify visually whether the patient is being treated with antibiotics or steroids at ICU admission. In addition, there are a large variety of uses for antibiotics and steroids. Because prophylactic use of antibiotics [17-19] and MPSS therapy for spinal cord injury [20] are still controversial therapies, these indications may change in the future. Furthermore, even if treatment with antibiotics and steroid administration were evaluated along with the two factors of intubation and open wound, the sensitivity for acquisition of MRSA infection would be almost the same as that for the two factors alone.

We excluded the patients with preexisting MRSA on ICU admission because the purpose of this study was to identify the risk factors for healthcare-associated (nosocomial) MRSA infection in the patients who did not harbor MRSA at the time of ICU admission. Our goal was to identify the patients vulnerable to MRSA infection. We think this is very important from the viewpoint of nosocomial infection control. Patients with preexisting MRSA on ICU admission are an important group because they are a source of MRSA and of development of infection. We also separately investigated predictors of infection for patients with preexisting MRSA (data not shown). The number of patients in this study with MRSA on ICU admission was 19 patients, and of them, 9 patients (47%) developed infection during their ICU stay. Due to this small number of patients, we analyzed the risk factor of MRSA infection by univariate analysis alone, and only 'transferred from another hospital' was detected as a significant risk factor. We have already introduced contact precautions for transferred patients until their MRSA status can be proven to be negative by surveillance culture.

We acknowledge several limitations in this study. First, the sample size was small because the study duration was only 1 year. Second, this study was carried out in a single institution. Because there is epidemiologic variation in healthcare-associated infection among institutions, results potentially may not be universally applicable. Third, external validation was not performed in this study. It was difficult to divide the patients to developing and validating data set due to the small sample size of patients with MRSA infection (n = 30) in this study. In the future, it will be necessary to perform an external validation analysis or to examine whether preemptive infection control for patients with high-risk factors actually works effectively. Fourth, patients intubated after the first 24 hours from ICU admission were not investigated. Because those patients will also be at high risk for MRSA infection in terms of disease severity and contact incidence with medical workers, they should also be targeted for preemptive infection control. Our preliminary study warrants further multicenter investigation, and we are presently in the process of conducting a prospective multi-institutional cohort study to assess the effect of focused preemptive infection control according to risk factors for HA-MRSA infection revealed in the present study.

## Conclusions

In the present study, we analyzed risk factors for HA-MRSA infection in an ICU from data obtained within 24 hours of patient ICU admission. Consequently, four prognostic variables were selected: intubation, open wound, treatment with antibiotics, and steroid administration, all occurring within 24 hours of ICU admission. Patients with the two factors of intubation or open wound included nearly 100% of the patients infected with MRSA, whereas patients with these factors were limited to about one half of all admissions to the ICU. Further investigation of the effectiveness of preemptive infection control to reduce HA-MRSA infection in these selected patients in the ICU setting is required.

## Competing interests

The authors declare that they have no competing interests.

## Authors' contributions

KY participated in study design and in data collection and interpretation, performed the statistical analysis and drafted the manuscript. OT had a major impact on the interpretation of data and critical appraisal of the manuscript. MF and JK participated in medical coworker's education and data collection. HM, YN and SF participated in data interpretation. HO, YK and TS conceived the study and its design and helped to draft the manuscript. TH performed the statistical analysis and helped to draft the manuscript. All the authors read and approved the final manuscript.

## Pre-publication history

The pre-publication history for this paper can be accessed here:

http://www.biomedcentral.com/1471-2334/11/303/prepub
